# CorkOakDB—The Cork Oak Genome Database Portal

**DOI:** 10.1093/database/baaa114

**Published:** 2020-12-31

**Authors:** Cirenia Arias-Baldrich, Marta Contreiras Silva, Filippo Bergeretti, Inês Chaves, Célia Miguel, Nelson J M Saibo, Daniel Sobral, Daniel Faria, Pedro M Barros

**Affiliations:** Instituto Gulbenkian de Ciência, Rua da Quinta Grande, Oeiras 2780-156, Lisboa, Portugal; Department of Biological and Medical Sciences, Oxford Brookes University, Headington Campus, Oxford OX3 0BP, UK; Instituto de Tecnologia Química e Biológica António Xavier, Universidade NOVA de Lisboa, Av. da República, Oeiras 2780-157, Lisboa, Portugal; Instituto de Tecnologia Química e Biológica António Xavier, Universidade NOVA de Lisboa, Av. da República, Oeiras 2780-157, Lisboa, Portugal; Instituto de Tecnologia Química e Biológica António Xavier, Universidade NOVA de Lisboa, Av. da República, Oeiras 2780-157, Lisboa, Portugal; Instituto de Biologia Experimental Tecnológica (iBET), Av. da República, 2780-157 Oeiras, Lisboa, Portugal; Instituto de Biologia Experimental Tecnológica (iBET), Av. da República, 2780-157 Oeiras, Lisboa, Portugal; Biosystems & Integrative Sciences Institute (BioISI), Faculdade de Ciências, Universidade de Lisboa, Campo Grande, Lisboa 1749-016, Portugal; Instituto de Tecnologia Química e Biológica António Xavier, Universidade NOVA de Lisboa, Av. da República, Oeiras 2780-157, Lisboa, Portugal; Instituto Gulbenkian de Ciência, Rua da Quinta Grande, Oeiras 2780-156, Lisboa, Portugal; UCIBIO, Departamento de Ciências da Vida, Faculdade de Ciências e Tecnologia, Universidade NOVA de Lisboa, Campus de Caparica, Caparica 2825-149, Setúbal, Portugal; Instituto Gulbenkian de Ciência, Rua da Quinta Grande, Oeiras 2780-156, Lisboa, Portugal; INESC-ID- Instituto de Engenharia de Sistemas e Computadores, Investigação e Desenvolvimento, Rua Alves Redol, Lisboa 1000-029, Portugal; Instituto de Tecnologia Química e Biológica António Xavier, Universidade NOVA de Lisboa, Av. da República, Oeiras 2780-157, Lisboa, Portugal

## Abstract

*Quercus suber* (cork oak) is an evergreen tree native to the Mediterranean basin, which plays a key role in the ecology and economy of this area. Over the last decades, this species has gone through an observable decline, mostly due to environmental factors. Deciphering the mechanisms of cork oak’s response to the environment and getting a deep insight into its biology are crucial to counteract biotic and abiotic stresses compromising the stability of a unique ecosystem. In the light of these setbacks, the publication of the genome in 2018 was a major step towards understanding the genetic make-up of this species. In an effort to integrate this information in a comprehensive, accessible and intuitive format, we have developed The Cork Oak Genome Database Portal (CorkOakDB). The CorkOakDB is supported by the BioData.pt e-infrastructure, the Portuguese ELIXIR node for biological data. The portal gives public access to search and explore the curated genomic and transcriptomic data on this species. Moreover, CorkOakDB provides a user-friendly interface and functional tools to help the research community take advantage of the increased accessibility to genomic information. A study case is provided to highlight the functionalities of the portal. CorkOakDB guarantees the update, curation and data collection, aiming to collect data besides the genetic/genomic information, in order to become the main repository in cork oak research.

**Database URL:**  http://corkoakdb.org/

## Introduction

Cork oak woodlands are unique and emblematic resources in the Mediterranean region, with high economic, ecological and social significance. They are natural ecosystems harbouring a wide range of biodiversity that act as large carbon sinks and protect against soil erosion and desertification, in addition to supporting local economies. The longevity and high activity of the cork cambium from cork oak are the cornerstones of the sustainable exploitation of a unique raw material with a wide range of industrial applications and high commercial value. Despite the great value of cork and the remarkable survival capacity of this species, cork oak stands are in decline in the Mediterranean region, due to both abiotic and biotic factors (1–3). Thus, it is increasingly critical to amplify research on this species and acquire the fundamental knowledge needed to develop strategies towards improved yield and resilience, which can ensure the conservation of this important agrosilvopastoral system.

Fundamental research in forest tree species is hindered by several factors, including their typically large and complex genomes, their long lifespan and the lack of publicly available tools to support research. Despite these challenges, multiple data sets have been released over the last decade covering different biological aspects of cork oak. Namely, in 2014, a consortium of Portuguese institutions released the CorkOakDB (4), which hosted the first reference transcriptome for cork oak, based on Expressed Sequence Tags (ESTs). This was obtained through 454 pyrosequencing of normalized cDNA libraries covering multiple organs, tissues, developmental stages and experimental conditions (4). Additionally, several comparative transcriptomic studies on cork oak have also been published in the meantime (5–10). More recently, in 2018, the first draft genome of cork oak was publicly released (11) providing a pivotal tool for genomic studies in the species. The predicted size of the first draft genome is 953.3 Mbp and organized in 23 344 scaffolds, with 94.6% of the genome represented in 4730 scaffolds larger than 10 kbp (11). The structural annotation available at NCBI (CorkOak1.0, GCF_002906115.1) includes a total of 58 326 genes and 59 614 transcripts with complete open reading frames.

While all the data mentioned above are already present in central public databases, they are distributed across numerous independent data sets. Creating an integrated repository devoted to cork oak omics is paramount to leverage all the genomic and transcriptomic data available and foster research on this species. Indeed, the centralization of genomic information about particular species in single repositories has proven a valuable tool for research on those species, as evidenced by their numerous citations (12–14).

This paper reports on the development and release of a major refactoring of the CorkOakDB to incorporate the draft genome of cork oak and recent transcriptomic data. This new release allows an integrated view of the genome, with browsing, sequence retrieval and gene expression visualization functionalities. In this manuscript, we provide a brief overview of existing technology for developing genome portals and further describe the CorkOakDB portal’s architecture, data and functionalities. Finally, we describe a case study showcasing the use of the portal for practical applications and discuss the impact of the portal for further genomic studies in cork oak.

## Technical specifications

Organism-specific genome portals have existed since the dawn of the internet and remain an important resource for data integration, community gathering, and fostering research and collaboration (15). The core functionalities of genome portals relate to the visualization of genomic data and corresponding annotations, enabling sequence-based searching. With respect to visualizing genomic data, JBrowse has become one of the standard solutions in the genomics community, as it offers the capacity to visualize and browse genomic features and to easily integrate into websites (16). Regarding sequence-based searching, the Basic Local Alignment Search Tool (BLAST) remains a standard solution for both organism-specific and general-purpose sequence databases (17). BLAST includes different algorithms such as blastn for nucleotide/nucleotide searches; blastx for nucleotide/protein searches; tblastn for protein/nucleotide searches; blastp for protein/protein searches and other variants of these algorithms more attuned for finding distant homologues.

One thing that has changed since the first genome portals arose is the availability of website content management systems and toolkits such as the Tripal (‘Tripal | Tripal’, https://tripal.info/, accessed 15 June 2020) framework (18), which greatly facilitates the development of new portals. Tripal is an open-source toolkit for biological databases that is part of the Generic Model Organism Database project tools and is the basis of several genome portals (13, 14). It includes BLAST and JBrowse modules, as well as modules for gene expression and functional annotation visualization. Tripal is built on top of Drupal (‘Drupal’, https://www.drupal.org/, accessed 15 June 2020), a content management system that provides a framework for the maintenance and administration of web portals, including functionalities such as user management, page editing and configuring menu structure. Tripal consists of a suite of Drupal modules integrated with Chado (19), a relational database schema aimed towards storing genomic data using the postgreSQL database management system. Chado stores genome sequences, gene expression data, functional annotation data and their attributes, as well as information on biological samples, publications and analysis, among others. Biological sequences representing genes, exons, transcripts or polypeptides are stored in Chado as (genomic) features.

With respect to experimental metadata, the core sequencing databases have adopted the BioProjects and BioSamples metadata schema (20), with BioProjects describing the experimental setting and research project and BioSamples describing the biological materials and their collection and processing. As organism-specific portals typically integrate data that are also deposited in core sequencing databases, they tend to implicitly adopt this metadata schema.

## The CorkOakDB portal

The CorkOakDB was developed using Tripal (18). In addition to the standard built-in Tripal modules, CorkOakDB is equipped with Tripal Extension Modules dedicated to data analysis, annotation and visualization, such as Tripal Analysis Expression, Tripal Analysis BLAST (17), Tripal Analysis InterPro and third-party Integration Extension Modules, including Tripal JBrowse—used for the integration of a pre-installed GMOD JBrowse instance (16)—that extend the functionality and possibilities of the portal.

### Data

CorkOakDB integrates *Quercus suber* genomic and transcriptomic data and their functional annotations and indexes relevant scientific publications on this species. Genomic data were obtained from the cork oak genome assembly deposited on NCBI (CorkOak1.0, GCF_002906115.1) and include structural annotation (in GFF format) and predicted transcript and protein sequences (in FASTA format). The identification of genomic features in the database follows the unique ID system from NCBI for CorkOak1.0 assembly. This uses a prefix specifying the type of biological sequence (e.g. polypeptide, gene and RNA) followed by the motif LOC, XP or XM and a unique identifier number.

**Figure 1.**
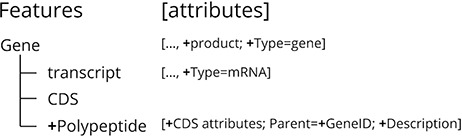
Summary of the new features added to the structural annotation file from the *Q. suber* genome publication for the CorkOakDB, which are identified with ‘+’.

The structural annotation file was modified to improve clarity and facilitate data retrieval for the end user. These modifications maintained both structure and integrity of the standard format while complementing the connections between feature IDs to facilitate navigation between feature types (gene, transcript and polypeptide). The major modification in the structure was the addition of the feature polypeptide as a child term of the correspondent gene feature ID (Figure 1 and Figure S1), to enable searches by polypeptide data. A product tag was also included in the Attributes column of the gene feature, to capture the information present in the corresponding product tags from their transcript child terms. These adjustments to the annotation file defined the hierarchical relationship between all features represented in the portal (gene, mRNA and polypeptide) and included product information in gene features that was previously only present in mRNA and polypeptide features.

The Attributes column for gene, mRNA, CDS (coding sequence) and polypeptide features in the annotation file was further populated with a description tag, in which functional information and alternative IDs, retrieved from previous publications regarding specific genes, were added as a string. This provided a connection with GenBank accession numbers of *Q. suber* gene sequences deposited before the genome sequence release. The correspondence between the former and the latter was established using blastp searches. Functional information was retrieved from corresponding publications (when available) and added to the Description tab using controlled vocabularies.

Additionally, *Q. suber* protein sequences were mapped to Interpro (IPR) domains and gene ontology (GO) terms using a local installation of InterProScan (version 5.35-74.0). The InterProScan output files were then loaded into the portal using the loader provided by the InterPro module added to CorkOakDB.

**Figure 2.**
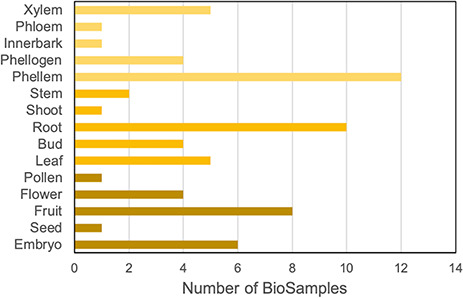
Distribution of cork oak publicly available NCBI BioSamples related to RNA-Seq data sets, according to cork oak tissue or organ.

To integrate gene expression data in the portal, we collected all available RNA-Seq data for *Q. suber* in the Sequence Read Archive (SRA) public repository (21). We identified 15 relevant BioProjects, which, as summarized in Figure 2, span a variety of organs, tissues and developmental stages, thereby providing a comprehensive representation of the cork oak transcriptome (4–11, 22–25). Also, some of the samples have undergone treatments such as heat/cold stress and drought and biotic stress, which add layers of information to the CorkOakDB. These data can be of great relevance for users working in the field, as they enable the screening of genes that play a role in biological processes of interest, such as development or response to stress.

Most of the data sets retrieved from SRA were obtained from non-normalized cDNA libraries, but two data sets [PRJEB6178—Acorn Development (6) and PRJEB3237—ESTs (4)] included sequencing data from normalized cDNA libraries. This type of library preparation produces a more even coverage of genes, which inherently affects the accuracy of the gene expression estimation within each library. However, since these BioProjects include multiple tissues, the estimated expression could provide clues about the activity of specific genes in different tissues (e.g. presence or absence of expression). Therefore, we decided to include these data sets in the portal, while providing the necessary information regarding library normalization in the BioProjects metadata page (available in the About menu). This area gathers all metadata retrieved from SRA that was manually curated to complete missing fields.

The *Q. suber* data sets retrieved from SRA were produced with two different high-throughput sequencing platforms: Roche 454 GS System and Illumina Genome Analyzer. Data sets from the two platforms were processed differently, taking into consideration the fact that Illumina data sets were already pre-processed (adaptors, empty reads and low-quality sequences were removed before uploading to the public repository) whereas 454 data sets were raw. Four hundred and fifty-four data sets were therefore processed with TagCleaner (v0.16) (26) and Trimmomatic (v0.38) (27) to remove adapters and low-quality bases, respectively.

All data sets were mapped to the reference genome with either GMAP (v2018-07-04) (28) or HISAT2 (v2.1.0) (29) for 454 or Illumina data sets, respectively. Read counts as transcripts per million (TPM) were obtained using StringTie (v1.3.5) (30). The resulting expression data were formatted according to the specifications required by the Tripal Analysis Expression module (31). Thus, matrix files were produced with biosample names in the first row and unique gene IDs in the first column and uploaded to the portal using the Expression Data Loader.

Publication data in the CorkOakDB are automatically mined and retrieved from the PubMed database (‘PubMed’, https://pubmed.ncbi.nlm.nih.gov/, accessed 6 July 2020) using Chado Bulk Publication Importers. A first importer was created to search for publications that included ‘Cork Oak’, ‘*Quercus suber*’ or ‘*Q. suber*’ in either the title or abstract. Once this initial import was done, a second importer was created to constantly update the publications, searching for new scientific papers matching the search criteria, every 15 days. The importer automatically retrieves the publications that match the criteria and adds them to the database.

Homologues of cork oak mRNA sequences in other plant species were identified by BLAST (17) searching against the ExPASy SwissProt (32) protein database using a local BLAST installation. These results were then added to the portal using the appropriate loader from the module and can be accessed in individual transcript content pages (see ‘Interface and functionalities’ section).

CorkOakDB’s architecture enables the update of data without significant alterations to the structure of the portal, and new genomic and biological data can be uploaded directly at any time. In this way, as the community grows and more projects become available, the resulting information can be promptly included, keeping the portal up to date. The standard organization of other static pages will also allow the easy update of the information included.

### Interface and functionalities

The CorkOakDB user interface conforms with the standard interfaces of established genome portals, being intuitive to use for researchers acquainted with these types of portals. The main menu options—About, Search, and Tools—create a clear distinction of functionalities, directing users for information about CorkOakDB, search menus or other tools available. Additionally, all menus and tools include explanations on how to use them, so that users unfamiliar with these types of portals can make effective use with little learning effort.

#### About menu

The About menu provides a general overview of the portal, including pages with a brief overview of the organism and the methodology used for the assembly of the draft genome, the BioData.pt research infrastructure, responsible for the portal, and the GENOSUBER consortium, responsible for the cork oak genome sequencing. The BioProjects page contains metadata with specific information related to each BioProject included in the portal, which was manually curated according to the MIAPPE v1.1 standard (33). Additionally, we included a page with external links to other platforms, such as the Breeding API (34) and other tree genomic databases, as well as the previous version of the CorkOakDB.

#### Search and individual content pages

**Figure 3.**
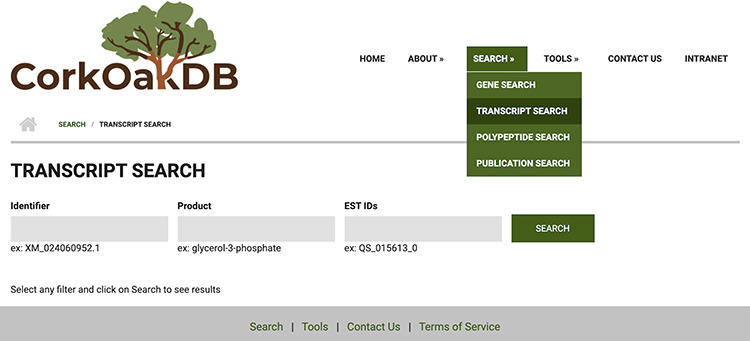
View of Transcript Search page in CorkOakDB.

The search engines are a central component of the portal. With these, the user can perform gene, transcript and polypeptide searches using corresponding IDs or keywords, or publication search of peer-reviewed scientific work on cork oak. Searches for these content types include several filters that can be applied to narrow down the results. Users can also use cork oak EST IDs to search for the corresponding transcript match from the draft genome (Figure 3). Clicking on an item in the results list will take the user to an individual page for that feature, which has different expandable fields that gather all related information.

For each type of content (publications, genes, polypeptides, transcripts, analysis, organism and biological samples), the individual page has several expandable fields containing all the information available in the portal about that specific record. However, not all pages contain the same level of information. Pages for publications, biological samples and analyses are static and contain only a summary table. Feature pages (genes, transcript and polypeptide) have dynamic fields to access sequences, annotation coordinates, parent/child IDs and gene expression. For example, from a gene page, the user can learn its annotation coordinates, nucleotide sequence, the predicted transcript and polypeptide product IDs, and expression values in specific RNA-Seq data sets.

**Figure 4.**
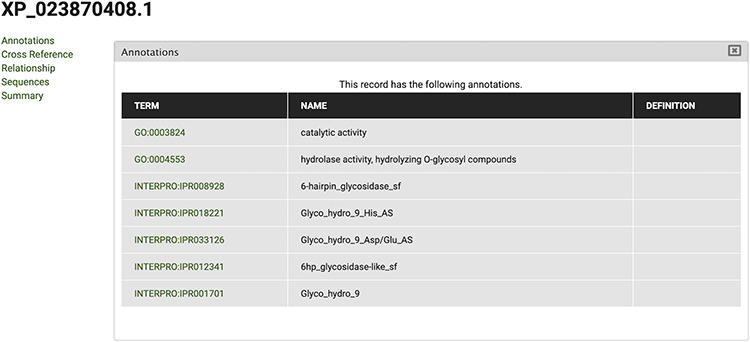
View of InterPro and Gene Ontology annotations for polypeptide sequence XP_023870408.1, obtained by selecting the ‘Annotations’ tab in the CorkOakDB.

**Figure 5.**
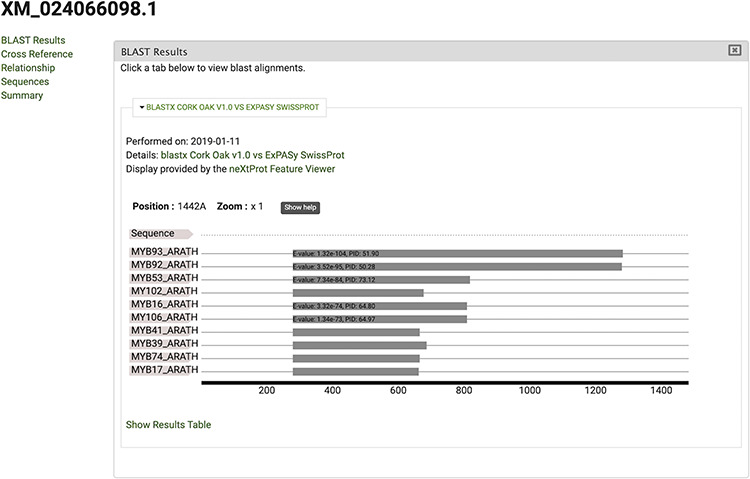
View of significant blastx hits for mRNA sequence XM_024066098.1, obtained by selecting the ‘BLAST results’ tab.

The gene feature page includes an expandable field for visualizing gene expression results in individual expression graphs, which is one of the functionalities enabled by the Tripal Analysis: Expression module (Tripal | Tripal Analysis: Expression, https://github.com/tripal/tripal_analysis_expression, accessed 23 June 2020). Within the expression, graph users can select an analysis, and the displayed biological samples can be sorted and coloured according to several attributes. This allows the user to compare the expression of the same gene across all BioSamples available in the portal. Moreover, transcript and polypeptide feature pages include functional annotation. Polypeptide pages include InterProScan annotations (Figure 4), and transcript pages include BLAST results in their table of contents (Figure 5). SwissProt matches redirect the user to the SwissProt website. Finally, the Publication pages include corresponding metadata for a given publication, including title, authors, abstract and cross-reference to the corresponding PubMed source.

#### Tools

Through the tools menu, users have access to complementary tools for data retrieval and analysis, namely homology search with BLAST (17), genome browsing with JBrowse (16) and gene expression data through the generation of heatmaps. Furthermore, links to download genome sequence (FASTA) and annotation (GFF) files are also provided.

BLAST allows users to search the cork oak genome for sequences homologous to an input sequence of interest, either introduced in the text box or uploaded as a FASTA file. The Tripal BLAST extension module allows users to use BLAST against preloaded nucleotide, protein and genomic databases. CorkOakDB includes three BLAST databases that the user can select in the BLAST options: a protein database containing all cork oak proteins, a nucleotide database containing all cork oak mRNA sequences and a genomic database containing assembled scaffolds. Users can also select one of the four available BLAST algorithms (blastn, blastx, tblastn or blastp) and configure the query with advanced options.

**Figure 6.**
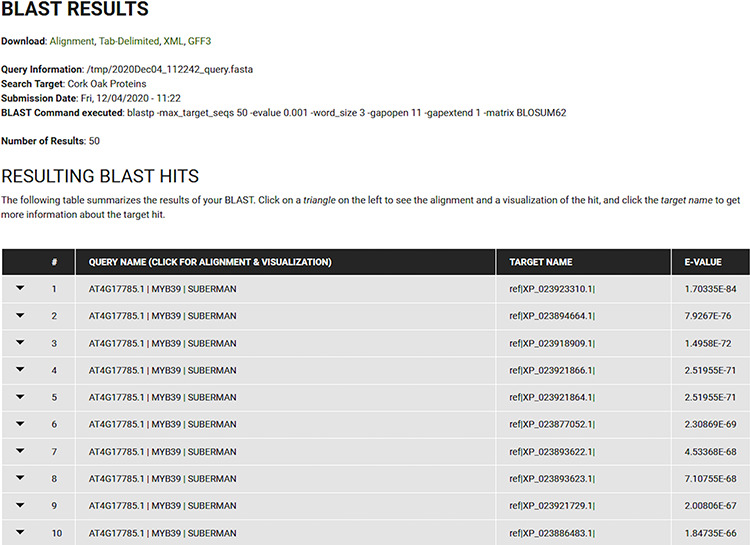
BLAST results for blastp search of the *Arabidopsis* MYB39 transcription factor on the cork oak protein database displayed in CorkOakDB.

The resulting BLAST hits are presented in a standard table (Query Name, Target Name and E-value) as collapsible fields that can be further explored, as depicted in Figure 6. Each field includes the information specific to the corresponding hit and includes a visual representation of the relationship between query and target, and the alignment. Users can also download the results in various formats (Alignment, Tab-Delimited, XML or GFF3).

**Figure 7.**
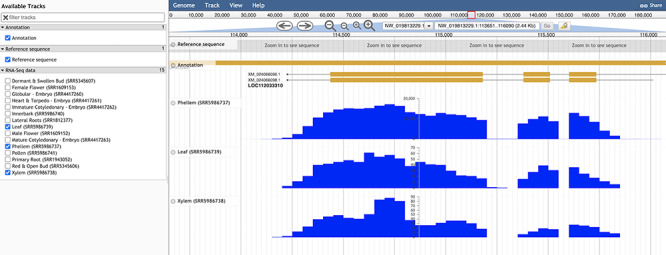
Genome browser view of LOC112033310 structural annotation. RNA-seq data from three different tissues were selected to showcase exon coverage.

With the Genome Browser tool (35), a user may visualize the cork oak genome, having access to genome feature annotations along the multiple scaffolds, in addition to optional tracks related to RNA-Seq data, as displayed in Figure 7. When using this tool, the user has the option to select any of the available tracks and features, after which the module will show a graphic display of the sequences and data sets selected. There are several options available to the user for manipulation of the display, such as scroll, zoom, search and enabling or disabling tracks. For more specific customization, the tracks can be further configured within the option ‘Edit track configuration’. There is also the option of learning more about this track, such as scores and number of bases covered, that can be accessed through the option ‘About this track’.

**Figure 8.**
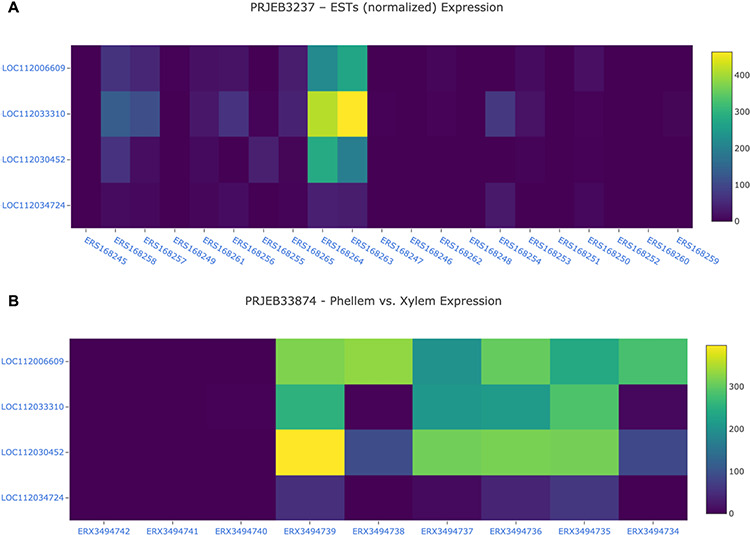
Gene expression analysis for the cork oak *MYB39* (LOC112034724) and *MYB92* (LOC112033310) orthologues, and two closely related MYB genes (LOC112030452 and LOC112006609) in different BioSamples included in BioProjects PRJEB3237 (A) and PRJEB33874 (B). These BioProjects were selected since they represent different cork oak tissues. BioSamples ERS168264, ERS168263 (upper panel), and ERX3494734 to ERX3494739 were obtained from developing phellem (or cork). BioSample names are hyperlinks to retrieve corresponding metadata.

The Heatmap tool was build using the Tripal Analysis: Expression module. This tool enables the user to visualize the expression of a list of genes, selected by their unique names, and/or GO and InterPro terms. In the resulting display (Figure 8), a specific BioProject can be selected and an option to sort the display by several attributes is available. This module is particularly useful when several genes are being studied as a group, as the heatmap enables users to visually represent the expression values of all genes of interest across different biological samples.

Finally, under Direct Downloads, users can download the data contained in the portal in bulk, including the genomic, protein and mRNA FASTA files, as well as the structural annotation (GFF file) and additional files containing functional annotation (e.g. InterPro and Gene Ontology).

## Case study

To demonstrate the use of CorkOakDB, we detail a case study of identifying cork oak candidate genes related to cork development.

The unique physical properties described for cork are mainly due to the high abundance of a natural biopolymer embedded in cork cell walls, called suberin. Suberin is also present in plant cell walls from interface tissues in other plant species, playing an important role in defence against external stresses (biotic or abiotic). The molecular mechanisms regulating cork suberization are still poorly described in cork oak, but this knowledge could be useful to design novel strategies to improve cork development and quality. AtMYB39 (SUBERMAN) and AtMYB92 are two *Arabidopsis* MYB transcription factors (TFs) recently identified as key regulators of suberin synthesis (36, 37).

**Figure 9.**
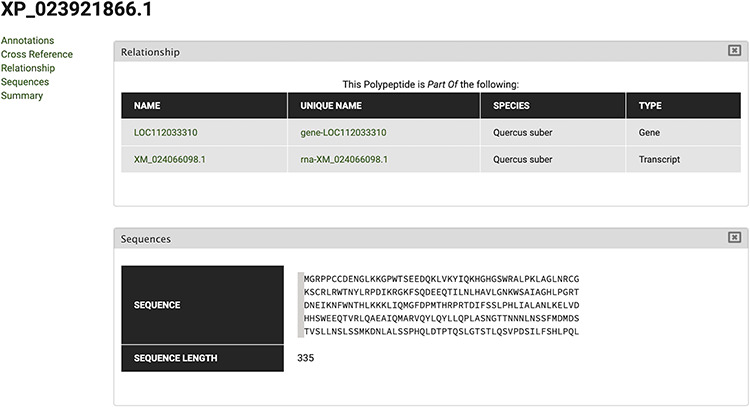
Polypeptide feature page for XP_023921866 polypeptide. Using the Relationship tab, the correspondent Gene and Transcript IDs can be retrieved.

**Figure 10.**
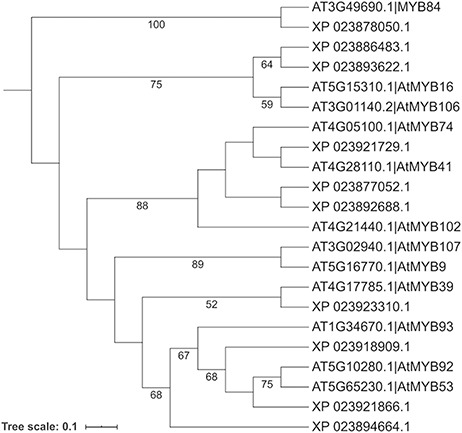
Phylogenetic analysis of selected cork oak and *Arabidopsis* MYBs TFs related to AtMYB39 and AtMYB92. Amino acid sequences were retrieved from CorkOakDB and TAIR, and multiple alignment of conserved MYB domains was performed using MAFFT v7. Phylogenetic inference was obtained using the Maximum Likelihood method with RAxML v8.2.12. Branch support was obtained by bootstrap analysis (1000 replications) and indicated for specific nodes (bootstrap value >50%).

To search for the cork oak orthologues of AtMYB39 and AtMYB92, we performed a blastp search of the correspondent polypeptide sequences (AT4G17785 and AT5G10280) against the cork oak protein database, using the BLAST tool from CoakOakDB (Figure 6 and Table S1). The best BLAST hits for AtMYB39 and AtMYB92 were XP_023923310.1 and XP_023921866.1/XP_023921864.1, respectively. The two protein IDs related to MYB92 correspond to different gene structural annotations (putative alternative splicing forms). Based on the top 10 hits obtained for AtMYB39 and AtMYB92 searches, we observed that nine hits were common in both analyses (Table S1), suggesting a close phylogenetic relationship, as also predicted for *Arabidopsis* (38). After performing individual polypeptide searches by ID, we retrieved the predicted amino acid sequences and gene IDs for each hit using the Sequences and Relationship tabs (Figure 9). To assess the phylogenetic relatedness of the selected cork oak MYBs, we conducted a phylogenetic analysis including *Arabidopsis* MYB TFs closely related to AtMYB39 and AtMYB92 (using the classification reported by Dubos *et al*. (2010). Phylogenetic inference based on the conserved MYB domain grouped XP_023923310.1 (LOC112034724) and AtMYB39, while XP_023921866.1 (LOC112033310) was included in a clade containing AtMYB92 and AtMYB53 (Figure 10).

For a preliminary assessment of the putative function of LOC112034724 and LOC112033310 and other related cork oak MYBs, we assessed the transcript abundance on specific gene expression data sets, using the Heatmap analysis tool. We selected two gene expression data sets containing multiple cork oak tissues, including developing cork—PRJEB3237 and PRJNA392919 (Figure 8). Interestingly, expression of these genes was enriched in developing cork (Figure 8A, sample IDs ERS168263 and ERS168264; Figure 8B, sample IDs ERX3494734 to ERX3494739), suggesting their involvement in suberin synthesis and/or cork development. LOC112034724 (*MYB39* candidate orthologue) is the gene showing the lowest expression in cork tissue samples, yet the TPM value is higher than in other samples (tissues) from the same data set. The identified cork oak MYBs are therefore candidate players in cork development and may be further targeted for functional characterization, which would require a detailed knowledge of intron and exon structure. We used the Genome Browser tool to obtain the structural annotation of LOC112033310 using the correspondent gene coordinates (NW_019813229.1:114240.133444) retrieved from the gene profile page. We confirmed the presence of two annotated transcripts resulting in the two protein products initially identified (Figure 7). The confidence of the exon annotations was also assessed by displaying the global read coverage obtained from RNA-Seq data for specific tissues, selected from the left panel. The two transcript isoforms differ mostly in the size of the 5ʹ untranslated region, but this difference would require further experimental validation.

This simple workflow using exclusively the tools and data sets available in CorkOakDB showcases its usefulness for genomics tasks such as gene function prediction. It demonstrates the merits of having a centralized portal that integrates all publicly available data to facilitate and foster research.

## Conclusions

CorkOakDB aims to be a reference hub for research on *Q. suber* by aggregating all available genomic and transcriptomic data on this species and offering a set of standard tools for data visualization and retrieval that enable core genomics analyses such as candidate gene identification and selection for functional studies, as demonstrated in our case study.

We will continue updating the portal contents to ensure that users have access to the latest data available. The addition of other data sets, reflecting improvement in genome sequence assembly and annotation, and transcriptomic changes occurring in specific developmental stages will contribute to increase the value and completeness of the portal. Furthermore, other individual cork oak trees are likely to be sequenced in the future, using the genome sequence now available as a reference, and including these novel data in the portal will allow the identification of genetic variability related to genes of interest.

Cork oak is a crucial species to the Portuguese economy and identity, with ongoing efforts for its improvement, management and conservation. Studying the genetic structure of cork oak is essential for the success of these efforts, which require identification and study of genes involved in traits of interest, such as cork production or response to biotic and abiotic challenges. CorkOakDB is therefore a pivotal tool which will greatly contribute to the success of these efforts.

## Supplementary Material

baaa114_Supp
