# Supplementary material for: CorkOakDB—The Cork Oak Genome Database Portal
Source: Database (Oxford). 2020 Dec 31;2020:baaa114. doi: 10.1093/database/baaa114 (PMC7774466; doi:10.1093/database/baaa114)
Supplement: baaa114_Supp [file baaa114_supp.zip › SupplementalMaterial.docx]

CorkOakDB - The Cork Oak Genome Database Portal

Cirenia Arias-Baldrich^1,5^*, Marta Contreiras Silva^2^*, Filippo Bergeretti^2^, Inês Chaves^2,3^, Célia Miguel^3,4^, Nelson Saibo^2^, Daniel Sobral^1,6^, Daniel Faria^1,7^**, Pedro M. Barros^2^**

# **Supplemental Material**

**Supplemental Table S1.** Top 10 BLASTp hits and corresponding E-value obtained for AtMYB39 and AtMYB92 homology searches on cork oak predicted proteins database. The gene ID and description was further retrieved using the CorkOakDB Polypeptide Search menu.

|  | **BLAST Hit** | **E-value** | **Gene ID** | **Description** |
| --- | --- | --- | --- | --- |
| **MYB39**  (AT4G17785) | XP_023923310.1 | 1.703E-84 | LOC112034724 | transcription factor MYB41 |
|  | XP_023894664.1 | 7.927E-76 | LOC112006609 | transcription factor MYB53 |
|  | XP_023918909.1 | 1.496E-72 | LOC112030452 | transcription factor MYB93 |
|  | XP_023921866.1 | 2.519E-71 | LOC112033310 | transcription factor MYB93-like |
|  | XP_023921864.1 | 2.519E-71 |  |  |
|  | XP_023877052.1 | 2.308E-69 | LOC111989495 | transcription factor MYB102-like |
|  | XP_023893622.1 | 4.534E-68 | LOC112005568 | transcription factor MYB16-like |
|  | XP_023893623.1 | 7.107E-68 |  |  |
|  | XP_023921729.1 | 2.008E-67 | LOC112033181 | transcription factor MYB102-like |
|  | XP_023886483.1 | 1.847E-66 | LOC111998628 | transcription factor MYB106-like |
| **MYB92**  (AT5G10280) | XP_023921866.1 | 2.749E-104 | LOC112033310 | transcription factor MYB93-like |
|  | XP_023921864.1 | 2.749E-104 |  |  |
|  | XP_023918909.1 | 4.245E-99 | LOC112030452 | transcription factor MYB93 |
|  | XP_023894664.1 | 4.356E-87 | LOC112006609 | transcription factor MYB53 |
|  | XP_023923310.1 | 3.591E-81 | LOC112034724 | transcription factor MYB41 |
|  | XP_023877052.1 | 1.806E-72 | LOC111989495 | transcription factor MYB102-like |
|  | XP_023892688.1 | 3.287E-72 | LOC112004686 | transcription factor MYB41-like |
|  | XP_023893622.1 | 1.683E-71 | LOC112005568 | transcription factor MYB16-like |
|  | XP_023893623.1 | 2.439E-71 |  |  |
|  | XP_023921729.1 | 2.676E-71 | LOC112033181 | transcription factor MYB102-like |

**
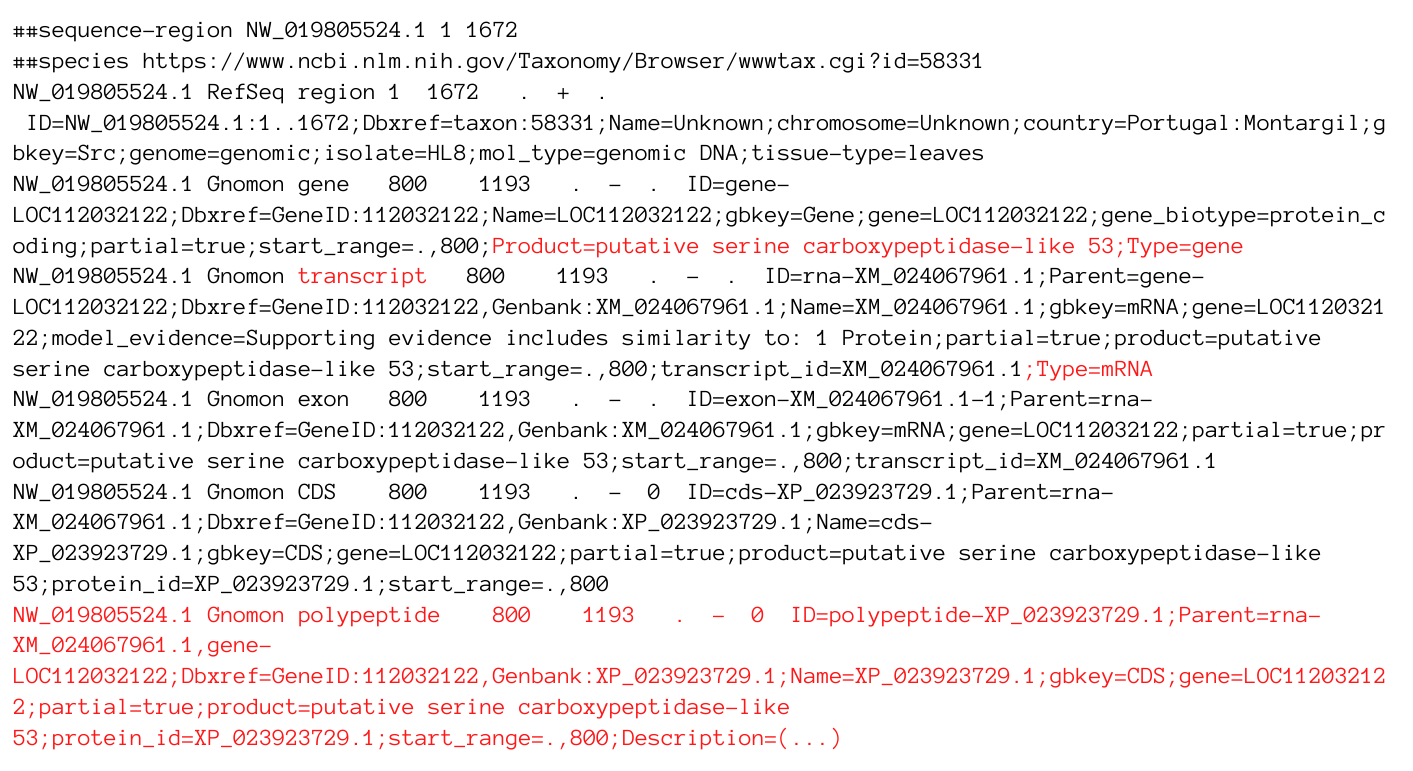
**

**Supplemental Figure S1**. Example from the annotation file with structural modifications highlighted in red.
